# Supplementary material for: Towards reduction in bias in epidemic curves due to outcome misclassification through Bayesian analysis of time-series of laboratory test results: case study of COVID-19 in Alberta, Canada and Philadelphia, USA
Source: BMC Med Res Methodol. 2020 Jun 6;20:146. doi: 10.1186/s12874-020-01037-4 (PMC7275354; doi:10.1186/s12874-020-01037-4)
Supplement: Supplementary file 1 — Additional file 1. [file 12874_2020_1037_MOESM1_ESM.docx]

**Towards reduction in bias in epidemic curves due to outcome misclassification through Bayesian analysis of time-series of laboratory test results:** Case study of COVID-19 in Alberta, Canada and Philadelphia, USA

**Supplementary Material**

[**Appendix A: Data used in the manuscript** 2](#_Toc37237596)

[Table A1: Timeline of counts of positive and negative tests for COVID-19 and cases per onset date in Alberta, Canada on 3/28/2020 2](#_Toc37237597)

[Table A2: Timeline of positive and negative tests for COVID-19 in Philadelphia, USA; obtained from figures published by City of Philadelphia on 3/31/2020 4](#_Toc37237598)

[**Appendix B: Statistical Modeling Details** 5](#_Toc37237599)

[**Appendix C: R code for Figures E and F** (code to produce panel as .png files is omitted) 6](#_Toc37237600)

## **Appendix A: Data used in the manuscript**

### Table A1: Timeline of counts of positive and negative tests for COVID-19 and cases per onset date in Alberta, Canada on 3/28/2020

| **Lab report date /date of onset for incident case (t)** | **test positive (Y_t_^*^)^a^** | **test negative (n_t_- Y_t_^*^)** | **Observed incident cases of COVID-19 (C_t_^*^)^a^** |
| --- | --- | --- | --- |
| 2020-03-06 | 1 | 61 | 1 |
| 2020-03-07 | 2 | 275 | 0 |
| 2020-03-08 | 3 | 440 | 0 |
| 2020-03-09 | 6 | 718 | 6 |
| 2020-03-10 | 6 | 966 | 9 |
| 2020-03-11 | 5 | 961 | 7 |
| 2020-03-12 | 2 | 1144 | 2 |
| 2020-03-13 | 8 | 1419 | 8 |
| 2020-03-14 | 19 | 1591 | 19 |
| 2020-03-15 | 9 | 1784 | 10 |
| 2020-03-16 | 20 | 1306 | 24 |
| 2020-03-17 | 8 | 2137 | 15 |
| 2020-03-18 | 27 | 2860 | 28 |
| 2020-03-19 | 31 | 2774 | 29 |
| 2020-03-20 | 31 | 3392 | 45 |
| 2020-03-21 | 26 | 3539 | 40 |
| 2020-03-22 | 29 | 2613 | 45 |
| 2020-03-23 | 36 | 2713 | 53 |
| 2020-03-24 | 50 | 3131 | 62 |
| 2020-03-25 | 51 | 1842 | 77 |
| 2020-03-26 | 31 | 1121 | 48 |
| 2020-03-27 | 11 | 593 | 14 |

^a^ the number of persons who tested positive at time t is not the same as number with persons with onset of symptoms at time t (used in epidemic curve); the lack of agreement two counts and their totals is due to lag in reporting and investigation of date of onset, a situation that is expected in the middle of epidemic at the time of compilation of these data

### Table A2: Timeline of positive and negative tests for COVID-19 in Philadelphia, USA; obtained from figures published by City of Philadelphia on 3/31/2020

| **date of test report (t)** | **test positive (Y_t_^*^)** | **test negative (n_t_- Y_t_^*^)** |
| --- | --- | --- |
| 3/10/2020 | 1 | 5 |
| 3/11/2020 | 0 | 13 |
| 3/12/2020 | 1 | 19 |
| 3/13/2020 | 2 | 46 |
| 3/14/2020 | 6 | 47 |
| 3/15/2020 | 1 | 28 |
| 3/16/2020 | 7 | 79 |
| 3/17/2020 | 15 | 157 |
| 3/18/2020 | 8 | 173 |
| 3/19/2020 | 30 | 267 |
| 3/20/2020 | 32 | 175 |
| 3/21/2020 | 50 | 358 |
| 3/22/2020 | 51 | 178 |
| 3/23/2020 | 72 | 387 |
| 3/24/2020 | 104 | 564 |
| 3/25/2020 | 164 | 725 |
| 3/26/2020 | 176 | 626 |
| 3/27/2020 | 192 | 973 |
| 3/28/2020 | 122 | 650 |
| 3/29/2020 | 153 | 614 |
| 3/30/2020 | 101 | 647 |
| 3/31/2020 | 27 | 99 |

## **Appendix B: Statistical Modeling Details**

Let r_k_ be the infection prevalence in the testing pool on the day corresponding to the k-th of K knots, and let Sn_k_ be the test sensitivity achieved within this pool. Without ambiguity, when we index with t to indicate the t-th of T days (as opposed to k for the k-th of K knots), r_t_ and Sn_t_ to be the prevalence and sensitivity achieved via linear interpolation from the straddling knots. Let Sp be the test specificity, assumed to be constant in time.

A prior for prevalence is specified as a distribution for r_1:K_ with independent uniform components. The left and right endpoints for each component then comprise 2K hyperparameters, though in our applications we reduce this to a single hyperparameter, by specifying a common upper-bound while setting zero as a common lower-bound.

A prior for sensitivity is constructed by taking Sn_k_ = (1-w)Sn_A,k_ + w Sn_B,k_. Here the prior specification for Sn_A:1:K_ is via independent uniform components with the hyperparameters being the corresponding 2K endpoints. The second component is perfectly linear, so that only (Sn_B,1_ , Sn_B,K_) are stochastic, taken as independently and uniformly distributed using the same endpoints as for the corresponding components of Sn_A_. Thus, when w=0 the prior for Sn has the same structure (but different hyperparameters) as the prior for r. As w increases, however, the time trajectories for Sn become smoother. We complete the specification by assigning a prior to w itself, namely w ~ Unif(0.5, 0.9). Thus, we do not arbitrarily dictate the trajectory smoothness *a priori*. Note also that in our applications we use common lower and upper bounds for Sn_1:K_, so that only two hyperparameters are needed.

Having defined a prior for r, Sn, and Sp, we now specify a model for the observed number of positive tests on the t-th day, $Y_{t}^{*}$, out of n_t_ tests. We build this up from a factorization of the form

f($Y_{t}^{*}$,Y_A,t_ , Y_B,t_ , Y_t_ | r_t_ , Sn_t_, Sp) = f($Y_{t}^{*}$|Y_A,t_ , Y_B,t_ )f(Y_A,t_ , Y_B,t_ | Y_t_ , Sn_t_, Sp) f(Y_t_ | r_t_ ).

Here Y_t_ ~ Bin(n_t_ , r_t_) is the true number of day t positives, which is ultimately the parameter of interest. Whereas t-th observed count decomposes $Y_{t}^{*}$= Y_A,t_ + Y_B,t_, where Y_A,t_ ~ Bin(Y_t_, Sn_t_) is the portion of the observed count arising as true positives, while independently Y_B,t_ ~ Bin(n_t_ - Y_t_, 1-Sp) is the portion arising as false positives.

The above comprises a complete stochastic generative model. Hence, Markov chain Monte Carlo sampling from the joint distribution of all latent quantities given all observed quantities is feasible. The latents include w, Sn_A:1:K_, (Sn_B,1_ , Sn_B,K_), Sp, r_1:K,_ Y_1:T,_ Y_A,1:T_ , and Y_B,1:T_. The observed data are the two series: $Y_{1:T}^{*}$and n_1:T_.

Implementation of the model is easily accomplished using the JAGS program ([mcmc-jags.sourceforge.net](http://mcmc-jags.sourceforge.net)), run from the R package rjags ([cran.r-project.org/web/packages/rjags](https://cran.r-project.org/web/packages/rjags)). JAGS simply requires the user to specify a complete generative model. The user does not need to specify what combinations of MCMC updates are applied to yield a Monte Carlo sample of all latent variables given all observed variables. The general strategy of JAGS is to use Gibbs sampling, whereby the multivariate target distribution is update one component at a time. However, the implementation details on how each such update is manifested are opaque to the user. Our implementation code is publicly available ([github.com/paulgstf/misclass-covid-19-testing](https://github.com/paulgstf/misclass-covid-19-testing)).

To elaborate on the underidentified nature of the statistical problem, it is simplest to consider the case of Sp=1, in which case the observed data are governed by E($Y_{t}^{*}$)/n_t_ = r_t_ Sn_t_ so that indeed asking the data to completely distinguish Sn_t_ from r_t_ is impossible, at least in the absence of prior information. If the piecewise-linear assumptions on Sn_t_ and r_t_ are valid, however, then one might anticipate some partial information coming forward. Consider a single interval between two knots. If the data signal on this interval happens to be highly linear, then we have a severe paucity of information, as two coefficients describing the composite signal are being asked to inform about the four coefficients describing the two constituent signals, albeit with help from the prior bounds on the constituent signals. On the other hand, if the data signal happens to be quadratic, then we still face an underidentified situation, but at least now there are three pieces of information from the composite signal to partially inform the four descriptors on the constituent quantities.

## **Appendix C: R code for Figures E and F** (code to produce panel as .png files is omitted)

###################

#march 28, 2020 data from the Province of Alberta, Canada: 2020-03-06 to 2020-03-24:

#limit to presumably complete data little affected by lag in test results and diagnosis

#Figure E

###################

library(plotrix)

#Number of cases observed C*

cstr<-c(1,0,0, 6,9,7,2,8,19,10,24,15,28,29,45,40,45,53,62)

T<-length(cstr)

####################################################

#time-invariant SN (the left-hands side of Figure E)

####################################################

#observed epi curve

days<-rep(1:T)

#95% confidence interval for observed counts under Poisson distribution

#http://ms.mcmaster.ca/peter/s743/poissonalpha.html

li<-qchisq(0.025, 2*cstr)/2

ui<-qchisq(0.975, 2*(cstr+1))/2

plotCI(x=days,y=cstr,li=li,ui=ui,

col="blue", lwd=5, ylim=c(0,120), xlim=c(1,19),

ylab="predicted true cases (C)",

xlab="presumed date of onset (t: March 6-March 24, 2020)")

#used https://www.desmos.com/calculator/kx83qio7yl calculator to select param of beta dist for Sn

#adjusted epi curves MC sensitivity analysis

sim=10 #number of MC simulations

#SN=0.60

for (i in c(1:sim)){

sn=rbeta(T, 57.6,38.4)

y=cstr/sn

lines(days, y, col="black")

rm(y, sn)}

#SN=0.95

for (i in c(1:sim)){

sn=rbeta(T, 18.05,0.95)

y=cstr/sn

lines(days, y, col="lightgrey")

rm(y, sn)}

#SN=0.85

for (i in c(1:sim)){

sn=rbeta(T, 43.35,7.65)

y=cstr/sn

lines(days, y, col="red")

rm(y, sn)}

#SN=0.75

for (i in c(1:sim)){

sn=rbeta(T, 56.25,18.75)

y=cstr/sn

lines(days, y, col="orange")

rm(y, sn)}

legend(x=1, y=100, legend=c("Observed (C*) and 95%CI", "Sn=95%", "Sn=85%", "Sn=75%", "Sn=60%"),

col=c("blue", "lightgrey", "red", "orange", "black"), lty=c(1,1,1,1,1), lwd=c(5,1,1,1,1), bg="ivory", bty = "grey")

abline(h=seq(1,150,10),col="gray", lty=3)

####################################################

#time-varying SN (the right-hands side of Figure E)

####################################################

#observed epi curve

days<-rep(1:T)

sim=20 #number of simulation realizations plotted

#95% confidence interval for observed counts under Poisson distribution

#http://ms.mcmaster.ca/peter/s743/poissonalpha.html

li<-qchisq(0.025, 2*cstr)/2

ui<-qchisq(0.975, 2*(cstr+1))/2

plotCI(x=days,y=cstr,li=li,ui=ui,

col="blue", lwd=5, ylim=c(0,120), xlim=c(1,19),

ylab="predicted true cases (C)",

xlab="presumed date of onset (t: March 6-March 24, 2020)")

#adjusted epi curves MC sensitivity analysis

#SN increases 0.60 to 0.75 to .95

for (i in c(1:sim)){

sn1=rbeta(1+T/3, 57.6,38.4)

sn2=rbeta(T/3, 56.25,18.75)

sn3=rbeta(T/3, 18.05,0.95)

sn=c(sn1, sn2, sn3)

y=cstr/sn

lines(days, y, col="green")

rm(y, sn)}

#SN decreases 0.95 to 0.75 to ..6

for (i in c(1:sim)){

sn1=rbeta(1+T/3, 56.25,18.75)

sn2=rbeta(T/3, 18.05,0.95)

sn3=rbeta(T/3, 57.6,38.4)

sn=c(sn1, sn2, sn3)

y=cstr/sn

lines(days, y, col="brown")

rm(y, sn)}

legend(x=1, y=100, legend=c("Observed (C*) and 95%CI", "Sn increases from 60, 75, 95% at break-points",

"Sn decreases from 95, 75, 60% at break-points", "break-points"),

col=c("blue", "green", "brown", "black"), lty=c(1,1,1,3), lwd=c(5,1,1,1), bg="ivory", bty = "grey")

abline(h=seq(1,150,10),col="gray", lty=3)

abline(v=c(1+T/3, 2*T/3),col="black", lty=3)

#########end of code for Alberta##############

###################

#march 31, 2020 data from the city of Philadelphia, USA: 2020-03-10 to 2020-03-29:

#limit to presumably complete data little affected by lag in test results and diagnosis

#Figure F

###################

library(plotrix)

#Number of cases observed C*

cstr<-c(1,0,1,2,6,1,7,15,8,30,32,50,51,72,104,164,176,192,122,153)

T<-length(cstr)

####################################################

#time-invariant SN (the left-hands side of Figure F)

####################################################

#observed epi curve

days<-rep(1:T)

#95% confidence interval for observed counts under Poisson distribution

#http://ms.mcmaster.ca/peter/s743/poissonalpha.html

li<-qchisq(0.025, 2*cstr)/2

ui<-qchisq(0.975, 2*(cstr+1))/2

plotCI(x=days,y=cstr,li=li,ui=ui,

col="blue", lwd=5, ylim=c(0,400), xlim=c(1,T),

ylab="predicted true cases (C)",

xlab="presumed date of onset (t: March 10-March 29, 2020)")

#used https://www.desmos.com/calculator/kx83qio7yl calculator to select param of beta dist for Sn

#adjusted epi curves MC sensitivity analysis

sim=10 #number of MC simulations

#SN=0.60

for (i in c(1:sim)){

sn=rbeta(T, 57.6,38.4)

y=cstr/sn

lines(days, y, col="black")

rm(y, sn)}

#SN=0.95

for (i in c(1:sim)){

sn=rbeta(T, 18.05,0.95)

y=cstr/sn

lines(days, y, col="lightgrey")

rm(y, sn)}

#SN=0.85

for (i in c(1:sim)){

sn=rbeta(T, 43.35,7.65)

y=cstr/sn

lines(days, y, col="red")

rm(y, sn)}

#SN=0.75

for (i in c(1:sim)){

sn=rbeta(T, 56.25,18.75)

y=cstr/sn

lines(days, y, col="orange")

rm(y, sn)}

legend(x=1, y=200, legend=c("Observed (C*) and 95%CI", "Sn=95%", "Sn=85%", "Sn=75%", "Sn=60%"),

col=c("blue", "lightgrey", "red", "orange", "black"), lty=c(1,1,1,1,1), lwd=c(5,1,1,1,1), bg="ivory", bty = "grey")

abline(h=seq(1,500,25),col="gray", lty=3)

####################################################

#time-varying SN (the right-hands side of Figure F)

####################################################

#observed epi curve

days<-rep(1:T)

sim=20 #number of simulation realizations plotted

#95% confidence interval for observed counts under Poisson distribution

#http://ms.mcmaster.ca/peter/s743/poissonalpha.html

li<-qchisq(0.025, 2*cstr)/2

ui<-qchisq(0.975, 2*(cstr+1))/2

plotCI(x=days,y=cstr,li=li,ui=ui,

col="blue", lwd=5, ylim=c(0,400), xlim=c(1,T),

ylab="predicted true cases (C)",

xlab="presumed date of onset (t: March 10-March 29, 2020)")

#adjusted epi curves MC sensitivity analysis

#SN increases 0.60 to 0.75 to .95

for (i in c(1:sim)){

sn1=rbeta(7, 57.6,38.4)

sn2=rbeta(7, 56.25,18.75)

sn3=rbeta(6, 18.05,0.95)

sn=c(sn1, sn2, sn3)

y=cstr/sn

lines(days, y, col="green")

rm(y, sn)}

#SN decreases 0.95 to 0.75 to ..6

for (i in c(1:sim)){

sn1=rbeta(7, 56.25,18.75)

sn2=rbeta(7, 18.05,0.95)

sn3=rbeta(6, 57.6,38.4)

sn=c(sn1, sn2, sn3)

y=cstr/sn

lines(days, y, col="brown")

rm(y, sn)}

legend(x=1, y=350, legend=c("Observed (C*) and 95%CI", "Sn increases from 60, 75, 95% at break-points",

"Sn decreases from 95, 75, 60% at break-points", "break-points"),

col=c("blue", "green", "brown", "black"), lty=c(1,1,1,3), lwd=c(5,1,1,1), bg="ivory", bty = "grey")

abline(h=seq(1,400,25),col="gray", lty=3)

abline(v=c(1+T/3, 2*T/3),col="black", lty=3)

#########end of code for Philadelphia##############
